# Prevalence of COVID-19 Infection among Patients with Diabetes and Their Vaccination Coverage Status in Saudi Arabia: A Cross-Sectional Analysis from a Hospital-Based Diabetes Registry

**DOI:** 10.3390/vaccines10020310

**Published:** 2022-02-16

**Authors:** Ayla M. Tourkmani, Abdulaziz Mansour Bin Rsheed, Mohammad Saad AlEissa, Sulaiman Mohammed Alqahtani, Azzam Fahad AlOtaibi, Mohammed S. Almujil, Ibrahim H. ALKhashan, Turki N. ALNassar, Mansour N. ALOtaibi, Alian A. Alrasheedy

**Affiliations:** 1 Chronic Illness Center, Family and Community Medicine Department, Prince Sultan Military Medical City, Riyadh 11159, Saudi Arabia; aturkmany@psmmc.med.sa (A.M.T.); ambinrasheed@psmmc.med.sa (A.M.B.R.); maleissa@psmmc.med.sa (M.S.A.); smqahtani@psmmc.med.sa (S.M.A.); af.otaibi@psmmc.med.sa (A.F.A.); malmujil@psmmc.med.sa (M.S.A.); 2 College of Medicine, Imam Mohammad Ibn Saud Islamic University, Riyadh 13317, Saudi Arabia; ihialkhashan@sm.imamu.edu.sa; 3 Health Services, Ministry of Defense, Riyadh 12426, Saudi Arabia; tnalnassar@psmmc.med.sa (T.N.A.); motaibi@msd.med.sa (M.N.A.); 4 Department of Pharmacy Practice, College of Pharmacy, Qassim University, Buraidah 51452, Saudi Arabia

**Keywords:** COVID-19, diabetes, infections, vaccination, vaccines, vaccine hesitancy

## Abstract

Patients with diabetes have a higher risk of severe infection and mortality due to COVID-19. Considering the current limited effective pharmacological treatments, vaccination remains one of the most effective means to control the pandemic. The current study aimed to determine the prevalence of COVID-19 infection and the rate of COVID-19 vaccination coverage among patients with type 2 diabetes mellitus. The patients were identified from a diabetes hospital registry at Prince Sultan Military Medical City, Riyadh, Saudi Arabia in July 2021. The history of COVID-19 infection and the vaccination status were retrieved from the National Health Electronic Surveillance Network (HESN) program and the Seha platform, respectively. A total of 11,573 patients were included in this study (representing 99.5% of all patients in the registry). A total of 1981 patients (17.1%) had a history of confirmed COVID-19 infection. The rate of vaccination with a 1st dose was 84.8% (*n* = 9811), while the rate of full vaccination with the 2nd dose was 55.5% (*n* = 6422). The analysis showed that a higher proportion of male patients were fully vaccinated than female patients (61.0% versus 51.2%, *p* < 0.001). There were statistically significant differences among the age groups, with the full vaccination rate ranging from 59.0% for the 61–70-year-old age group to 49.0% for the > 80-year-old age group (*p* < 0.001). The patients with no previous history of COVID-19 infection were more likely to get fully vaccinated than those with a previous history of the infection (63.9% versus 14.6%, respectively, *p* < 0.001). The factors associated with a higher likelihood of unvaccinated status included the female gender (adjusted odds ratio (aOR) = 1.705 (95% confidence interval (CI): 1.528–1.902)), elderly patients in the age group of 61–70 (aOR (95% CI) = 1.390 (1.102–1.753)), the age group of 71–80 (aOR (95% CI) = 1.924 (1.499–2.470)) and the age group of >80 (aOR (95% CI) = 3.081 (2.252–4.214), and prior history of COVID-19 infection (aOR (95% CI) = 2.501 (2.223–2.813)). In conclusion, a considerable proportion of patients with type 2 diabetes had confirmed COVID-19 infection. Continued targeted efforts are needed to accelerate vaccination coverage rates among patients with diabetes in general and the particular subgroups identified in this study.

## 1. Introduction

The coronavirus disease 2019 (COVID-19) pandemic has affected all countries globally in many aspects of life since the severe acute respiratory syndrome coronavirus 2 (SARS-CoV-2) was identified in December 2019 and the subsequent global spread of this highly contagious virus [1,2,3]. Many countries suffered from several waves of COVID-19 with additional stress, pressure and strain on healthcare systems [4,5,6,7]. The basic reproduction number (R_0_) for COVID-19 was estimated to be in the range of 1.4 to 2.5, [2,8,9,10] with some studies reporting higher R_0_ (e.g., >6) during outbreaks in some areas or regions of the world [11,12]. This variability in the R_0_ estimates is due to several factors, including the model used for its estimation, public health interventions to control the pandemic, level of implementation of precautionary measures (e.g., travel restrictions, social distancing), and population size/density [12,13,14]. The case fatality rate (CFR) for COVID-19 ranges from 0.87% to 2.79% in different countries, with a global CFR estimate of approximately 2.07% [2,15]. Moreover, the morbidity and mortality rate of COVID-19 is higher among individuals with comorbid conditions and among older individuals [16,17,18,19,20]. As of 4 November 2021, the World Health Organization (WHO) has reported more than 247 million COVID-19 cases and more than 5.02 million deaths due to COVID-19 globally [21]. In Saudi Arabia, as of 4 November 2021, there were more than 0.5 million confirmed COVID-19 cases, resulting in 8799 deaths [22]. Similar to other countries, the Ministry of Health (MoH) in Saudi Arabia was actively engaged in their efforts to mitigate the COVID-19 pandemic by formulating public health interventions and society-level preventive measures and ensuring compliance with these preventive measures in collaboration with other authorities in Saudi Arabia [23,24,25,26,27]. Consequently, due to the effective strategies adopted in Saudi Arabia, the R_0_ decreased substantially [28].

Diabetes mellitus is a chronic condition resulting in high blood glucose levels either because of the failure of insulin secretion or action [29,30]. Globally, approximately 463 million individuals suffer from diabetes, and this figure is estimated to reach 700 million by 2045 [31]. Poor glycemic control among patients with diabetes increases their chances of getting infections; therefore, these individuals need to be more cautious than the general population [32,33,34]. Diabetes increases the severity and mortality rate of COVID-19 [35,36,37,38,39]. In a study conducted in Saudi Arabia, it was found that there is a higher prevalence of diabetes among admitted COVID-19 patients [40]. Additionally, it was also confirmed that patients with diabetes have higher mortality rate due to COVID-19 than nondiabetic patients [40]. Therefore, for patients with diabetes, compliance with preventive guidelines related to COVID-19 becomes even more important [41,42].

Considering that effective pharmacological treatment to combat COVID-19 is still emerging, vaccination has become an important pillar for controlling the COVID-19 pandemic [43,44,45]. As different types of vaccines have shown positive results and are now available for clinical use [3,46,47,48,49,50], it is quite important for patients with diabetes to receive vaccinations to avoid serious debilitating COVID-19 infection [51,52,53]. Pfizer-BioNtech was the first COVID-19 vaccine approved by the Saudi Food and Drug Authority (SFDA) on 10 December 2020 and was subsequently introduced in Saudi Arabia [54]. This was followed by the approval and introduction of the Oxford-AstraZeneca vaccine in Saudi Arabia on 18 February 2021 [55] and the Moderna vaccine on 11 July 2021 [56]. Consequently, three vaccines are currently available in Saudi Arabia, and more efforts are made to encourage vaccination of all eligible persons as soon as possible [57,58]. The national vaccination program for COVID-19 was launched on 17 December 2020 [59]. It consisted of three main stages with higher priority that were initially given to the most vulnerable groups of the population [60,61]. The 1st stage included target groups with higher priority, such as persons aged > 65 years old, professionals at higher risk of exposure to COVID-19 infection, obesity (BMI > 40), persons with immunodeficiency diseases or taking immunosuppressive medications, and patients with two or more chronic diseases (e.g., diabetes, hypertension, chronic kidney diseases, chronic heart disease). The second stage started on 18 February 2021 and included targeted groups such as persons > 50 years old, patients with any chronic disease, and obese individuals (BMI ≥ 30). The third stage included all persons interested in getting vaccinated [60,61,62]. The Ministry of Health emphasized that vaccines are free to all persons, including illegal residents. The vaccines were provided via a large number of centers and healthcare institutions in more than 587 centers throughout Saudi Arabia [59,61,63]. The access to vaccination centers and appointments for obtaining vaccines were made easier by e-booking services via Sehaty and Tawakkalna applications or by visiting any center of COVID-19 vaccines or health centers with vaccination services even without prior appointments, especially for elderly individuals and patients with comorbidities [59,61,63]. The timeline of the vaccination program is illustrated in Table 1.

Vaccine hesitancy, or the belief in conspiracy theories related to COVID-19 and misinformation about the COVID-19 pandemic and vaccines on social media have been important factors that impede COVID-19 preventive efforts, including timely vaccination coverage [64,65]. Other reasons for COVID-19 vaccine hesitancy include individuals’ concerns about the adverse effects of COVID-19 vaccines, lack of trust in COVID-19 vaccines, planning to wait and see if the vaccines are safe, believing that COVID-19 is not a serious disease, believing that there is no need for the vaccines or being not sure about their effectiveness [66,67]. Therefore, it is important for governments to work on educating the population against these conspiracy theories, as such theories could jeopardize the strategies to mitigate the spread of COVID-19 and prevent timely and wide vaccination coverage of the populations [64,68,69]. As of 17 September 2021, it was estimated that approximately 50% of the general population in Saudi Arabia was fully vaccinated against COVID-19 [70]. Studies have also been performed to identify the predictors of COVID-19 vaccination in Saudi Arabia [71,72,73]. These studies can be helpful in formulating an intervention to educate the public and reduce misconceptions and accelerate the vaccination coverage of the population [73]. Although it is helpful that the population-level vaccination coverage is estimated, the percentage of the individuals or groups of patients vulnerable to COVID-19 infections and its serious complications, such as patients with diabetes, are still not known. This is particularly important, as it is evident in the literature that some patients with diabetes in Saudi Arabia had received misinformation and developed irrational fears about COVID-19 vaccination [72]. A survey-based study conducted during March–May 2021 showed that only 34.7% of the study participants with diabetes had taken the COVID-19 vaccination [72]. Consequently, the aims of this study were to determine the prevalence of COVID-19 infection among patients with type 2 diabetes mellitus and to determine the rate of COVID-19 vaccination coverage among these patients using a large hospital-based diabetes registry of more than 11,500 patients with diabetes with a history of infection and vaccination status retrieved from unified national databases/platforms. Consequently, the findings of this study could provide useful data for health policy-makers and healthcare professionals to ensure timely vaccination coverage of vulnerable patients with chronic diseases and take the relevant interventions.

## 2. Patients and Methods

### 2.1. Study Design, Setting and Population

This was a cross-sectional study conducted on patients with type 2 diabetes mellitus. The patients with diabetes were identified and retrieved from the hospital diabetes registry at the Family and Community Medicine Department at Prince Sultan Military Medical City (PSMMC), Riyadh, Saudi Arabia. This registry included all patients with diabetes registered in the four chronic illness clinics at the PSMMC. The clinics are located in the middle, north, east and south regions of Riyadh city. The history of COVID-19 infection and vaccination coverage status were retrieved from the Saudi MOH platforms.

### 2.2. Sample Size and Method

The study employed a universal sampling method in which all patients in the PSMMC diabetes registry were targeted in this study. All patients with a national identification (ID) number in the registry were included. Consequently, patients with missing information in the registry, particularly the national ID number, were excluded. This is because the search in the Saudi MOH platforms (i.e., HESN and Seha) is only possible using the national ID number. As of 16 July 2021, there were over 11,500 patients with type 2 diabetes in the diabetes registry.

### 2.3. Data Sources and Collection Procedure

There were three data sources used in this study, namely, the PSMMC diabetes registry, Health Electronic Surveillance Network (HESN) and national vaccination record via the Seha platform. The data were collected from these three sources by trained specialized employees at PSMMC with authorized access to the databases and platforms. The data collection was performed over two weeks and was concluded on 16 July 2021. A brief description of the data sources and the procedure is summarized below.

#### 2.3.1. The PSMMC Diabetes Registry

All patients with type 2 diabetes had two key identification numbers, i.e., patient number and national ID number in the diabetes registry at PSMMC. The PSMMC diabetes registry was established in February 2019 to strengthen diabetes surveillance, manage patients with diabetes, and provide a support system for clinicians to make evidence-based decisions. These registry data can also be used to see the trends in the demographic and clinical characteristics as well as the outcomes of patients with diabetes over time. Initially, in February 2019, the diabetes registry used the data from only one chronic illnesses clinic and then expanded to include the data of other chronic illness clinics affiliated with family and community medicine at PSMMC in the Riyadh region. The PSMMC diabetes registry is fully electronic, using a web-based (Oracle) system. Consequently, from the registry, the patients with diabetes were identified and retrieved, including their national ID numbers, age, and sex.

#### 2.3.2. Health—Diseases Surveillance Network System (HESN)

Using the national ID number of the patients, the history of COVID-19 infection was retrieved from HESN. HESN is an official program established by the Saudi Ministry of Health. It is a Public Health—Diseases Surveillance and Management Solution (PH-DSMS) that acts as a one-stop platform that encompasses all public health information, including history of COVID-19 infections reported by all healthcare institutions in Saudi Arabia from the public and private sectors. To determine the history of infection with COVID-19, first, the national ID of the patients was extracted to an Excel sheet to enable the search in HESN to confirm whether the patients had COVID-19 infection, including relevant information such as the date of COVID-19 infection.

#### 2.3.3. National Vaccination Record via Seha Platform

To determine the vaccination status for each patient, the data were retrieved from the national vaccination record at MOH in Saudi Arabia via the Seha platform. Then, the relevant data, including vaccination status, number of doses administered (i.e., 1st dose, 2nd dose) and type of vaccination were recorded.

### 2.4. Data Management and Analysis

The data were initially entered into Microsoft Excel and then transferred to SPSS for Windows version 22. Descriptive statistics (e.g., frequencies and percentages) were used to summarize the data. Inferential statistics, including the Chi-squared test and logistic regression, were used to examine the associations between the variables. The statistical significance was set at a *p* value of <0.05.

## 3. Results

### 3.1. Sociodemographic Characteristics

A total of 11,573 patients with type 2 diabetes with complete identification data in the hospital-based diabetes registry were included in this study (60 patients were excluded because their relevant information could not be tracked due to missing information on their National ID number in the registry). Consequently, 99.5% of patients in the registry were included in the study. In terms of sex, 56.2% (*n* = 6506) were female, while 43.8% (*n* = 5067) were male. The mean age (standard deviation) of the patients was 57.71 (12.07), and the median (interquartile range) was 58 (50–65).

### 3.2. Prevalence of COVID-19 Infection among Patients with Type 2 Diabetes

Based on the data in the HESN, 17.1% (*n* = 1981) were infected with COVID-19. The rest of the patients showed no history of COVID-19 infection (82.9%; *n* = 9592). Furthermore, among patients with no history of infection with COVID-19, 31.1% (*n* = 3601) performed a test for COVID-19 (i.e., swab), and the results were negative, while 51.8% (*n* = 5991) had no history of infection or underwent tests for suspected COVID-19 infection.

### 3.3. Rate of Vaccination among Patients with Type 2 Diabetes Mellitus

**Figure 1:**
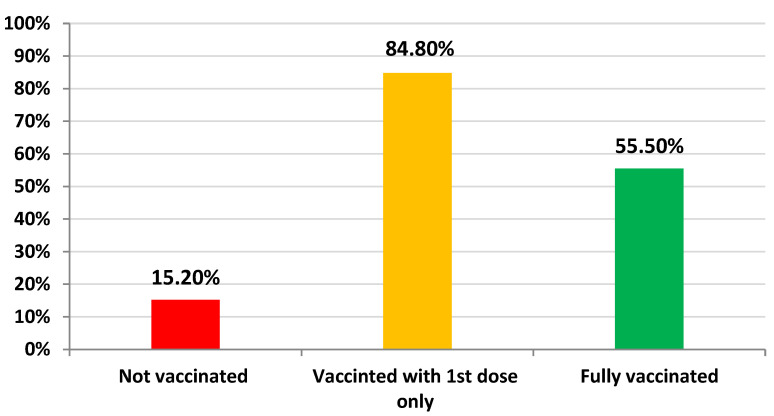
The rate of vaccination among patients with type 2 diabetes as of 16 July 2021.

The overall rate of vaccination with the 1st dose among patients with type 2 diabetes was 84.8% (*n* = 9811), while the rate of vaccination with the 2nd dose (i.e., fully vaccinated) was 55.5% (*n* = 6422). Consequently, in this study, 15.2% of the patients (*n* = 1762) were not vaccinated, and 44.5% (*n* = 5151) were not fully vaccinated (Figure 1). The types of vaccines administered to the patients were the Pfizer-BioNTech vaccine (64.9%; *n* = 7510) and Oxford/AstraZeneca vaccine (19.9%; *n* = 2301).

### 3.4. Association between Infection with COVID-19 and the Patients’ Sociodemographic Characteristics

There were no statistically significant associations between the infection rate and the patients’ age or sex (*p* < 0.05), as shown in Table 2. However, although not statistically significant, the elderly patients > 80 years old were at a higher risk of infection (i.e., 19.6%).

### 3.5. Association between Vaccination Status, the Patients’ Sociodemographic Characteristics and Previous Infection

The association between vaccination status (with at least the 1st dose of the vaccine) and the characteristics of the patients in terms of sex, age, and history of previous infection were examined. As shown in Table 3, there was a statistically significant association between the vaccination status and the sex of the patients. In particular, a higher proportion of male patients was vaccinated than female patients (88.5% versus 81.9%, *p* < 0.001). In terms of age, the higher the age of the patients, the less likely they were to be vaccinated, with a vaccination rate ranging from 88.7% for the age group ≤ 40 to 70.9% for the age group > 80 (*p* < 0.001). In this study, patients with no previous history of COVID-19 infection were more likely to get vaccinated than those with a previous history of the infection (87.1% versus 73.3%, respectively, *p* < 0.001).

### 3.6. Association between Full Vaccination Status, the Patients’ Sociodemographic Characteristics and Previous Infection

**Figure 2:**
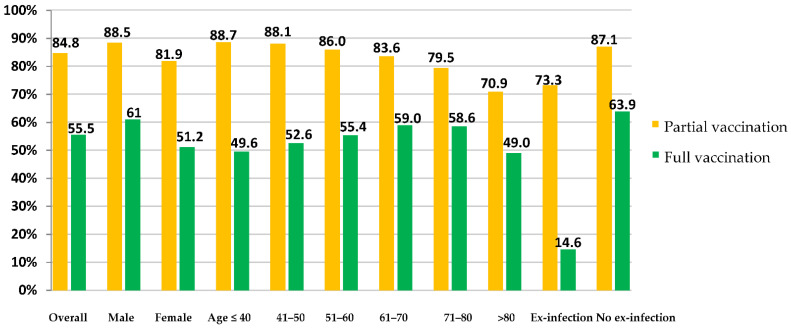
The rates of partial and full vaccination status among patients with type 2 diabetes as of 16 July 2021.

In this section, we examined the association between the full vaccination status (i.e., completed the two doses of the vaccine) and the characteristics of the patients in terms of sex, age, and history of previous infection. As shown in Table 4, there was a statistically significant association between the full vaccination status and the sex of the patients. In particular, a higher proportion of male patients were fully vaccinated than female patients (61.0% versus 51.2%, *p* < 0.001). In terms of age, there were statistically significant differences among the age groups. The full vaccination rate ranged from 59.0% for the 61–70-year-old age group compared to only 49.0% for the >80-year-old age group (*p* < 0.001). In this study, patients with no previous history of COVID-19 infection were more likely to get fully vaccinated than those with a previous history of the infection (63.9% versus 14.6%, respectively, *p* < 0.001). The rates of full and partial vaccination coverage among the different subgroups of patients are presented in Figure 2.

### 3.7. Logistic Regression Analysis for the Impact of Factors Associated with the Unvaccinated Status

As shown in Table 5, the logistic regression was conducted to further assess the impact of the sociodemographic characteristics on the status of being unvaccinated among the patients with type 2 diabetes mellitus, as there were 1762 patients who did not receive any vaccination for COVID-19. The findings confirmed the univariate analysis and have shown the subgroups with a higher likelihood of being unvaccinated. These included the female sex (adjusted odds ratio (aOR) = 1.705 (95% confidence interval (CI): 1.528–1.902)), elderly patients in the age group of 61–70 (aOR (95% CI) = 1.390 (1.102–1.753)), the age group of 71–80 (aOR (95% CI) = 1.924 (1.499–2.470)), and the age group of >80 (aOR (95% CI) = 3.081 (2.252–4.214). In addition, prior history of COVID-19 infection (aOR (95% CI) = 2.501 (2.223–2.813)) was statistically associated with being unvaccinated compared to those patients with no prior history of COVID-19 infection in the regression analysis.

## 4. Discussion

This is one of the few studies that reported the actual status of vaccination against COVID-19 among patients with diabetes and the actual rate of COVID-19 infection among this vulnerable group of patients. The previous studies in the literature were focused on determining the intentions of the general public, healthcare professionals or patients to get vaccinated (i.e., examining willingness, intentions of acceptance, hesitancy, etc. [82,83,84,85,86,87,88,89,90,91,92,93,94], with very few studies that assessed the vaccination status of particular groups of patients (e.g., cancer patients) [95].

The overall rate of confirmed COVID-19 infection was 17.1% in type 2 diabetes patients in this study. Moreover, it was alarming that elderly patients, especially patients aged > 80 years old, had a higher prevalence of COVID-19 infections than other patient age groups (19.6% became infected with COVID-19). This is a considerable proportion of this group of patients given the higher risk of severe infections with COVID-19 and its related complications in type 2 diabetes patients, including the risk of hospital admission, intensive care unit admission, morbidity and mortality [96,97,98]. Consequently, more education and awareness activities are needed to target patients with diabetes to help reduce the incidence of COVID-19 infections and subsequently the burden on the healthcare systems to manage its complications. This is because the relationship of COVID-19 and diabetes is bidirectional, as diabetes increases the severity of the infection, while COVID-19 could cause direct damage to the pancreas and worsen hyperglycemia or could even induce the onset of diabetes in previously nondiabetic individuals [97].

The rate of fully vaccinated patients with diabetes was only 55.5%, leaving a substantial number of individuals who were either not vaccinated or only partially vaccinated. This is less than expected and not consistent with the current COVID-19 vaccination program roll-out plan that was started in mid-December 2020 that aimed to ensure that almost all patients with comorbidities, including diabetes, be fully vaccinated by the time this study was conducted [59,61]. This is a lower-than-expected rate of vaccination coverage given that elderly patients and patients with chronic diseases, including diabetes, were given priorities to get vaccinated since the start of the program [59,61,62,74]. Vaccinations were available in more than 587 centers throughout Saudi Arabia. Moreover, vaccines are provided free to all patients and individuals and can be booked immediately through e-booking services via Sehaty and Tawakkalna applications or by just visiting any center of COVID-19 vaccines or health centers with vaccination services even without prior appointment, especially for elderly individuals and patients with comorbidities [63]. Consequently, based on the wide availability of vaccines, the large number of centers for vaccinations, all of which are free for all eligible individuals and are easily accessible through electronic booking and in-person appointments, including the priority given to the elderly and patients with diabetes, it is expected that the vast majority of elderly and patients with diabetes patients are fully vaccinated at the time of the study (Table 1). In fact, the COVID-19 vaccination program at the time of the study was at an advanced stage, as it reached the stage of vaccination of children in the age group of 12–18 years old in early July 2021 [77]. Lower vaccination coverage can be attributed to vaccine hesitancy, which has been reported in many countries, including Saudi Arabia [72,99,100]. Considering that COVID infection in patients with diabetes can result in debilitating and potentially life-threatening complications, it is important that vaccines be regularly advocated in individuals with diabetes mellitus to counter vaccine hesitancy and misconceptions due to conspiracy theories and other misinformation [64].

In this study, the status of full vaccination was associated with sex, age, and history of previous infection with COVID 19. Female patients had a lower rate of vaccination than male patients. Lower COVID-19 vaccination coverage in females compared to males is consistent with some other studies in the literature that showed a lower rate of COVID-19 vaccination [101] and a higher vaccine hesitancy among females [102]. A study from Maharashtra State, India, showed that only 84 women were vaccinated for every 100 men [101]. However, this is in contrast to the findings from the report on COVID-19 vaccination coverage among adults in the US that showed coverage with ≥1 dose COVID-19 vaccine was higher among females compared to males (58.0% versus 53.4%, respectively) [103]. Moreover, the lower vaccine uptake among the elderly patients in this study is concerning, as it has the potential to increase the risk of serious COVID-19 infections. The coverage with ≥1 dose COVID-19 vaccine is lower among the patients with age of ≥60 compared to the younger age groups. Moreover, a considerable proportion (29.1%) of elderly patients aged 80 and above was not vaccinated. This is in contrast to the situation in the US, in which elderly individuals and individuals aged ≥65 years had a higher rate of vaccination than other age groups [103]. Therefore, it is important to explore the reasons for this lower rate of vaccination among patients with diabetes in older age groups and among females through qualitative inquiries and formulate interventions to reduce this hesitancy, alleviate barriers and improve confidence in vaccines. It is important to educate the public about recent changes and updates in vaccination requirements. For example, in this study, a higher percentage of individuals with prior infection were not vaccinated compared to those with no history of infection. In addition, only 14.6% of patients with prior infection are fully vaccinated (i.e., received 2 doses). In Saudi Arabia, the MoH protocol requires patients infected with COVID-19 to get vaccinated (1 dose) 6 months after the infection. However, this was updated in late July 2021, and it was indicated that the vaccine could be taken 10 days after the infection and that two doses are now required regardless of any prior COVID-19 infection [80]. In addition, mixing of vaccines was approved in early July 2021 (i.e., the 2nd dose can be from a different vaccine than the initial one, e.g., if the 1st dose is Oxford/AstraZeneca, then the 2nd dose can be Pfizer/BioNtech vaccine) [76]. Therefore, it is of great importance to keep the public aware of the updates and information given the rapid developments in the science and clinical evidence related to COVID-19 and its vaccines.

The study has several strengths. This is one of the first studies that explored the vaccination coverage status and infection rate among patients with diabetes. Another strength is using a diabetes registry with a large number of patients (more than 11,500 patients with diabetes) visiting four different chronic illness clinics in different areas of Riyadh, Saudi Arabia. In addition, to reduce any potential bias due to the sampling method, all the patients in the registry were included and their data were used for the next steps to determine the vaccination status and infection rate. Moreover, the vaccination status and infection history were retrieved from reliable sources (i.e., the official records) and not based on self-reporting or measuring the intentions, etc. However, the study had some limitations. The morbidity and mortality outcomes from COVID-19 infection among patients with diabetes, such as the rate of hospital admission and intensive care unit admission were not determined. However, this was out of the scope of our specific objective of this study. The study was conducted at a particular point of time in July 2021 and reflected the rate of vaccination at that time. In addition, it was conducted in one city of Saudi Arabia, and consequently, it might not be generalizable to all diabetes patients in the country. However, the study findings are robust and could provide further guidance to health authorities and healthcare professionals to accelerate vaccination coverage to the vulnerable groups of patients.

## 5. Conclusions

A considerable proportion of patients with type 2 diabetes had confirmed COVID-19 infection. Consequently, more education and awareness of patients with diabetes about the evidence-based and correct use of preventive measures to avoid COVID-19 infection are of great importance. The vaccination coverage rate was lower than anticipated in this group of patients based on the set targets of the national vaccination plan at the time of the study. Therefore, continued targeted efforts are needed to accelerate vaccination rates among patients with diabetes in general and particular subgroups, including elderly patients, females and patients with prior COVID-19 infection. More efforts are needed to increase confidence in vaccines, alleviate barriers, and address any hesitancy or misinformation among patients. Healthcare professionals and clinicians providing diabetes care could play a vital role and address the importance of vaccination with their patients as part of their therapeutic plan.

## Figures and Tables

**Table 1:** Timeline and milestones of the national COVID-19 vaccination program in Saudi Arabia.

No.	Milestone	Date	Reference
1	Launching the national COVID1-19 vaccination program (1st stage)	17 December 2020	[59]
2	Announcement of the 2nd stage of the program	18 February 2021	[62]
3	Announcement of the administration of 2nd dose for ≥60	28 May 2021	[74]
4	Announcement of the administration of 2nd dose for ≥50	24 June 2021	[75]
5	Approval of mixing vaccines in Saudi Arabia	23 June 2021	[76]
6	Approval of Pfizer/BioNTech vaccine for ages 12–18	27 June 2021	[77]
7	Announcement of the administration of 2nd dose for ≥40	5 July 2021	[78]
8	Announcement of the administration of 2nd dose for all eligible persons of all ages	11 July 2021	[79]
9	Approval of receiving the vaccine after 10 days from recovery of COVID-19 infection (two doses regardless of the prior infection)	29 July 2021	[80]
10	Approval of Pfizer/BioNTech vaccine for the ages 5–11	3 November 2021	[81]

**Table 2:** Association between infection with COVID-19 and the patients’ characteristics.

Variable	History of COVID-19 Infection		*p* Value
	Yes (*n* = 1981) % (*n*)	No (*n* = 9592) % (*n*)	
Gender			0.817
Male	17.2% (872)	82.8% (4195)	
Female	17.0% (1109)	83.0% (5397)	
Age			0.098
≤40	18.6% (168)	81.4% (734)	
41–50	15.5% (340)	84.5% (1850)	
51–60	16.8% (651)	83.2% (3231)	
61–70	18.1% (528)	81.9% (2389)	
71–80	16.9% (224)	83.1% (1101)	
>80	19.6% (70)	80.4% (287)	

**Table 3:** Association between vaccination status and patient characteristics.

Variable	Vaccination Status (1st Dose Only)		*p* Value
	Yes (*n* = 9811) % (*n*)	No (*n* = 1762) % (*n*)	
Gender			<0.001
Male	88.5% (4482)	11.5% (585)	
Female	81.9% (5329)	18.1% (1177)	
Age			<0.001
≤40	88.7% (800)	11.3% (102)	
41–50	88.1% (1929)	11.9% (261)	
51–60	86.0% (3338)	14.0% (544)	
61–70	83.6% (2438)	16.4% (479)	
71–80	79.5% (1053)	20.5% (272)	
>80	70.9% (253)	29.1% (104)	
History of previous infection with COVID-19			<0.001
Yes	73.3% (1452)	26.7% (529)	
No	87.1% (8359)	12.9% (1233)	

**Table 4:** Association between full vaccination status and patient characteristics.

Variable	Fully Vaccination Status (Completed Two Doses)		*p* Value
	Yes (*n* = 6422) % (*n*)	No (*n* = 5151) % (*n*)	
Gender			<0.001
Male	61.0% (3091)	39.0% (1976)	
Female	51.2% (3331)	48.8% (3175)	
Age			<0.001
≤40	49.6% (447)	50.4% (455)	
41–50	52.6% (1153)	47.4% (1037)	
51–60	55.4% (2150)	44.6% (1732)	
61–70	59.0% (1721)	41.0% (1196)	
71–80	58.6% (776)	41.4% (549)	
>80	49.0% (175)	51.0% (182)	
History of previous infection with COVID-19			<0.001
Yes	14.6% (290)	85.4% (1691)	
No	63.9% (6132)	36.1% (3460)	

**Table 5:** Binary logistic regression for factors associated with unvaccinated status of the patients.

Variable	Adjusted Odds Ratio (95% CI)	*p* Value
Gender		
Male	1	*p <* 0.001
Female	1.705 (1.528–1.902)	
Age		*p* < 0.001
≤40	1	
41–50	1.035 (0.808–1.324)	0.786
51–60	1.155 (0.918–1.453)	0.219
61–70	1.390 (1.102–1.753)	0.006
71–80	1.924 (1.499–2.470)	*p* < 0.001
>80	3.081 (2.252–4.214)	*p* < 0.001
History of previous infection with COVID-19		
Yes	2.501 (2.223–2.813)	*p* < 0.001
No	1	

CI = Confidence interval.

## Data Availability

The data will be available from the corresponding author upon reasonable request.
